# Investigation of Molecular Structure and Thermal Properties of Thermo-Oxidative Aged SBS in Blends and Their Relations

**DOI:** 10.3390/ma10070768

**Published:** 2017-07-07

**Authors:** Xiong Xu, Jianying Yu, Lihui Xue, Canlin Zhang, Yagang Zha, Yi Gu

**Affiliations:** 1 State Key Laboratory of Silicate Materials for Architectures, Wuhan University of Technology, Wuhan 430070, China; xx900221@163.com (X.X.); zhangcanlin@whut.edu.cn (C.Z.); zhayagang1023@163.com (Y.Z.); 15608630308@163.com (Y.G.); 2 Center for Materials Research and Analysis, Wuhan University of Technology, Wuhan 430070, China

**Keywords:** SBS, thermo-oxidative aging, molecular structure, thermal properties

## Abstract

Tri-block copolymer styrene–butadiene (SBS) is extensively applied in bituminous highway construction due to its high elasticity and excellent weather resistance. With the extension of time, tri-block structural SBS automatically degrades into bi-block structural SB- with some terminal oxygen-containing groups under the comprehensive effects of light, heat, oxygen, etc. In this paper, the effects of aging temperature, aging time and oxygen concentration on the molecular structure of thermo-oxidative aged SBS were mainly investigated using Fourier transform infrared spectroscopy (FTIR) and X-ray photoelectron spectroscopy (XPS), and the correlation between oxygen-containing groups and thermal properties (TG–DTG) was further discussed. The FTIR and XPS results show that rapid decomposition of SBS will occur with increments of aging temperature, aging time and oxygen concentration, and a large number of oxygen-containing groups such as –OH, C=O, –COOH, etc. will be formed during thermo-oxidative aging. In short-term aging, changes in aging temperature and oxygen concentration have a significant impact on the structural damage of SBS. However, in long-term aging, it has no further effect on the molecular structure of SBS or on increasing oxygen concentration. The TG and DTG results indicate that the concentration of substances with low molecular weight gradually increases with the improvement of the degree of aging of the SBS, while the initial decomposition rate increases at the beginning of thermal weightlessness and the decomposition rate slows down in comparison with neat SBS. From the relation between the XPS and TG results, it can be seen that the initial thermal stability of SBS rapidly reduces as the relative concentration of the oxygen-containing groups accumulates around 3%, while the maximum decomposition temperature slowly decreases when the relative concentration of the oxygen-containing groups is more than 3%, due to the difficult damage to strong bonds on the molecular structure of aged SBS.

## 1. Introduction

Tri-block copolymer poly (styrene-b-butadiene-b-styrene) (SBS) has been widely applied in modified bitumen materials due to its excellent physical and mechanical properties [1,2,3,4,5,6]. It plays a vital role in improving the high and low temperatures of modified bitumen because of its unique molecular structure [7,8]. However, despite all this, the molecular structure will still suffer from the destruction from the outside environment no matter whether SBS exists in bitumen or not, as temperature, ultraviolet radiation and oxygen are major determinants leading to its structural failure [9,10,11,12,13]. Hence, large amounts of waste SBS-modified bitumen will be increasingly produced due to the performance deterioration of SBS after a long period of use [14,15,16,17,18,19,20,21].

**Figure 1:**
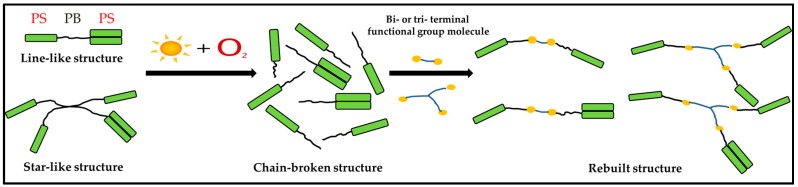
Reaction reconstruction process of poly (styrene-b-butadiene-b-styrene) (SBS).

For decades, the rejuvenation and recycling of materials has been a research hotspot in various fields [22,23,24,25]. In the domain of bitumen pavement, it is of great importance to solve the problem of the rejuvenation of waste SBS-modified bitumen. Until now, some literature has already reported that waste SBS-modified bitumen can be rejuvenated by adding traditional rejuvenators and/or fresh SBS-modified bitumen. While the properties of rejuvenated SBS-modified bitumen can be partially restored in this way, the recovery is only the result of an adjustment in the composition of the bitumen and/or the effect of fresh SBS-modified bitumen [26,27,28]. Therefore, the rejuvenation of aged SBS-modified bitumen should be focused not only on aged bitumen, but more on aged SBS. With respect to this problem, it is of great importance to investigate the molecular structure of aged SBS and explore the type and concentration of the newly-formed oxygen-containing groups, which can contribute to the structural reconstruction of SBS. As is known, oxygen-containing groups, such as hydroxyl and carboxyl groups, have some activity that can react with the epoxy group, isocyanate group, etc. On this basis, a design idea regarding the reaction rejuvenation of aged SBS using bi- or tri-terminal functional group molecules was considered. The reaction reconstruction process of SBS is shown in Figure 1.

Thermo-oxidative aging is an extremely important form resulting in structural damage and the performance deterioration of SBS [29,30,31]. Accordingly, some related researchers have so far investigated the effect of thermo-oxidative aging on the structure of SBS. For instance, Wang et al. studied the thermal oxidative degradation of SBS using FTIR, and found that polar hydroxyl and carbonyl groups appeared and carbon–carbon double bonds disappeared in the molecular chain of the SBS [32]. In addition, Munteanu et al. studied the thermo-oxidative behavior of SBS with different architectures, and discovered that the hydroxyl and carboxyl groups appeared on the molecular chain though FTIR [33]. To realize the reconstruction of SBS molecule, knowledge of the type and number of oxygen-containing groups in aged SBS in different conditions is, of course, essential in order to conduct a full exploration. So far, several related articles reported in publications have only qualitatively analyzed the structure and properties of aged SBS, however, the quantitative results have rarely been reported.

Due to the complexity of bituminous chemical composition, it is considerably difficult to extract aged SBS from bitumen mixture. In order to investigate the structure and properties of thermo-oxidative aged SBS in bitumen through a simulation, a high boiling-point solvent, naphtha, that can be easily removed, was selected for the present work. Besides this, the type and number of oxygen-containing groups and the concentration of carbon–carbon double bonds used to assess the aging degree of SBS were investigated using Fourier transform infrared spectroscopy (FTIR) and X-ray photoelectron spectroscopy (XPS). Furthermore, the thermal properties of SBS before and after aging were also investigated using a thermal gravimetric analyzer (TGA). Lastly, the relation between the oxygen-containing group concentration and the thermal stability of aged SBS was established.

## 2. Materials and Methods

### 2.1. Materials

Linear-like SBS (1301) with *M_n_* = 1 × 10^4^ g/mol, in which the mass ratio of styrene and butadiene is 30 to 70, was provided by Baling Petrochemical Co., Ltd (Yueyang, Hunan, China). Solvent. Solvent naphtha (200#), used as a substitute for bitumen, was supplied by Qilu Petrochemical Co., Ltd (Zibo, Shandong, China).

### 2.2. Extraction of Heavy Ends from Solvent Naphtha

Heavy ends in the temperature range of 200–230 °C were extracted from the solvent naphtha in the distilling apparatus at ambient temperature and pressure. All the heavy ends were prepared for solving SBS and conducting the higher temperature experiment.

### 2.3. Thermo-Oxidative Aging Procedure

The thermo-oxidative aging procedure of SBS in heavy ends was conducted in a self-made aging device equipped with an air-injection system. The concrete implementation process was as follows. Firstly, 400 mL SBS blends (SBS, by mass of 5%) were put in the aging device. Afterwards, the aging temperatures of 150 °C and 180 °C, the aging times of 3 h, 12 h and 48 h, and the oxygen flowrates of 0.25 L/min and 1 L/min were selected to investigate the effect of temperature, time and oxygen concentration on the structure and properties of SBS. Here, oxygen concentrations were controlled by the changing oxygen flowrate. At last, dried SBS in various degrees of aging was obtained at 50 °C for 72 h.

### 2.4. Fourier Transform Infrared Spectroscopy (FTIR)

Structural information about SBS before and after thermal aging was tested using a Nicolet 6700 FTIR spectrometer instrument (Thermo Fisher Scientific, Wellesley, MA, USA), with a maximum resolution of 0.019 cm^−1^, within the wavenumber range of 4000 and 400 cm^−1^. The tested resolution and scanning times were respectively set at 4 cm^−1^ and 64. Before testing, samples were first cast onto a kalium bromatum (KBr) pressed disk and then dried under vacuum conditions. 

Since the skeleton vibration of the benzene ring occurs in the range of spectral bands between 1600 and 1450 cm^−1^, in the short-term aging process there were no obvious changes. The aging degree of SBS was reflected using the relative functional group index calculated by Equation (1) [14,34]:

(1) $$I=\frac{\mathrm{Area}\;\mathrm{of}\;\mathrm{characteristic}\;\mathrm{absorbption}\;\mathrm{spectral}\;\mathrm{band}}{\sum^{\text{​}}\mathrm{Area}\;\mathrm{of}\;\mathrm{spectral}\;\mathrm{bands}\;\mathrm{between}\;1600\;\mathrm{and}\;1450{\;\mathrm{cm}}^{-1}}$$

### 2.5. X-ray Photoelectron Spectroscopy (XPS)

Information concerning the elements and structure of SBS before and after thermal aging was detected using a K-Alpha XPS (Thermo Fisher Scientific, Wellesley, MA, USA). Before detection, all the samples were coated onto the aluminum foil and pretreated in a vacuum environment for 24 h. Subsequently, the sample was tested with an irradiation of a mono-chromatic Al K_α_ X-ray source (1486.6 eV), with a spot area of 400 μm × 400 μm, energy step size of 1000 eV, and pass energy of 200 eV and 50 eV for survey scans and narrow scans, respectively. In addition, the binding energy peaks were calibrated with C1s at 284.8 eV as reference. The relative concentration of the elements and chemical groups were respectively calculated with the analytical software Avantage for quantitative analysis [35].

### 2.6. Thermal Analysis (TG-DTG)

TG and DTG are usually used to determine the thermal stability and decomposition rate of materials [36,37]. In this work, the thermal properties of SBS at various degrees of aging were investigated using a Netzsch STA449F3 thermal analyzer (Bavarian, Germany) with an inert nitrogen (N_2_) atmosphere. The tested temperature range and heating rate were set at 40–800 °C and 10 °C/min. Relevant information displayed on the TG and DTG curves was simultaneously obtained in the thermal decomposition process.

## 3. Results and Discussion

### 3.1. Effect of Aging Temperature on the Molecular Structure of SBS

**Figure 2:**
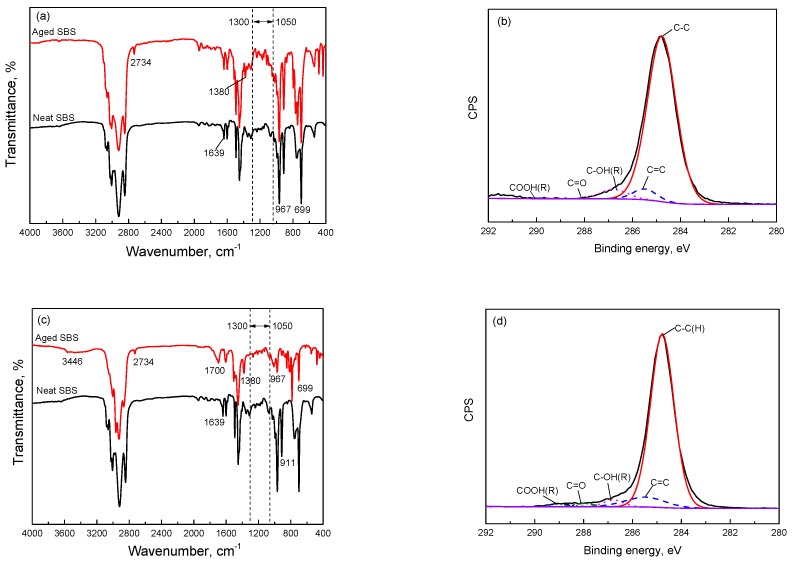
Fourier transform infrared spectroscopy (FTIR) and X-ray photoelectron spectroscopy (XPS) C1 spectra of (**a**,**b**) aged SBS exposure at 150 °C for 3 h with an oxygen flowrate of 1 L/min and (**c**,**d**) aged SBS exposure at 180 °C for 3 h with an oxygen flowrate of 1 L/min.

To investigate the effects of the thermo-oxidative temperature on the molecular structure of SBS, the structures and oxygen-containing groups after aging were characterized by FTIR and XPS, as shown in Figure 2. The time and oxygen flowrate were respectively 3 h and 1 L/min. Table 1 and Table 2, respectively, present the quantitative results of the relative functional group index and the relative concentrations of carbon related groups, in which the data for the neat SBS are from our previous research [38]. The results show that C=O and COOH(R) do not exist in the molecular structure of neat SBS and the concentration of C–OH(R) is low. After aging, oxygen-containing groups, such as hydroxyls, carbonyls, carboxyls, ethers, etc., have formed on the structure of the SBS. Compared with Figure 2a, Figure 2c shows the FTIR spectral peak signals at 3446 and 1700 cm^−1^ attributed to the stretching vibration of –OH and C=O; the other positions at 1639 and 967 cm^−1^ attributed to the stretching and bending vibration of C=C significantly decrease. Additionally, the peak intensity of C–OH(R), C=O and COOH(R) in the XPS C1s spectra (Figure 2d) become stronger than that in Figure 2b, which indicates that the higher temperature exposure causes a more serious oxidative reaction and exacerbates the formation of oxygen-containing reactive groups.

In Table 1 and Table 2, when compared with aged SBS (150 °C), we can see that the sum of I_C–OH(R)_ and I_C=O_ (from 0.038 to 0.108) and the relative concentrations of C–OH(R), C=O and COOH(R) (from 2.61% to 7.42%) were further improved at 180 °C, in particular, I_C–OH_ rose from 0 to 0.032. The results above indicate that more reactive hydroxyl groups were produced in the short-term, high-temperature aging procedure and the number of oxygen-containing carbonyl groups also increased with the rising aging temperature.

### 3.2. Effect of Aging Time on the Molecular Structure of SBS

**Figure 3:**
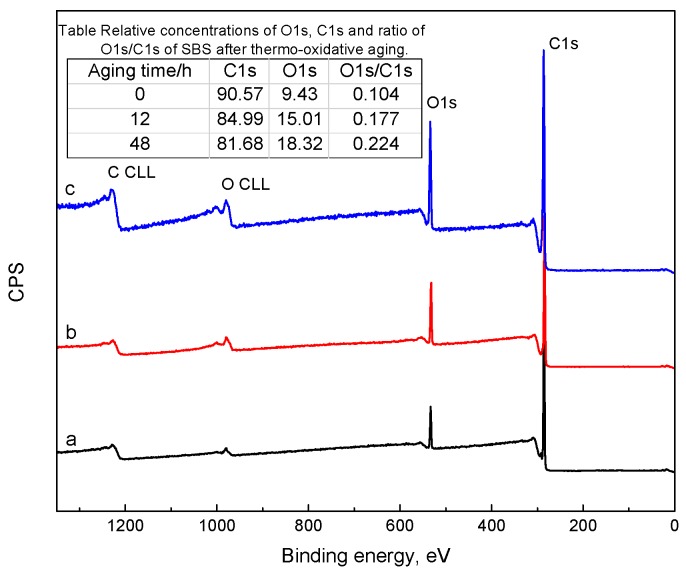
XPS survey spectra of (**a**) neat SBS; (**b**) aged SBS exposure at 150 °C for 12 h with an oxygen flowrate of 1 L/min; (**c**) aged SBS exposure at 150 °C for 48 h with an oxygen flowrate of 1 L/min.

**Figure 4:**
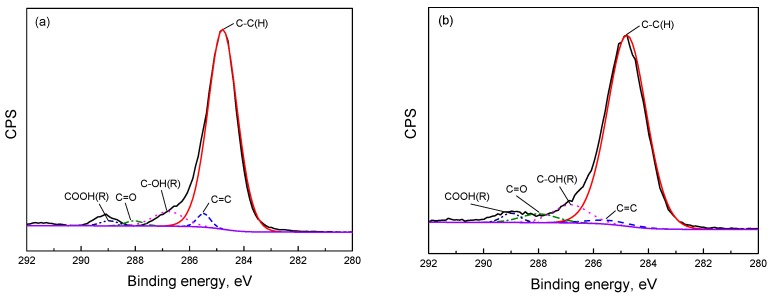
XPS C1s spectra of (**a**) aged SBS exposure at 150 °C for 12 h with an oxygen flowrate of 1 L/min and (**b**) aged SBS exposure at 150 °C for 48 h with an oxygen flowrate of 1 L/min.

According to the analysis above, the effect of a lower aging temperature on the molecular structure of SBS is not serious in the short-term aging procedure. Thus, the aging temperature of 150 °C was selected to investigate the effects of aging time on the molecular structure of SBS. Figure 3 and Table 3 show the XPS survey spectra and the relative concentration of carbon related to chemical groups of neat SBS, aged SBS (12 h) and aged SBS (48 h); the C1s spectra of aged SBS (12 h) and aged SBS (48 h) are also presented in Figure 4. It was found that the increased aging time enhanced the peak signal of O1s and weakened the peak signal of C1s, and that the ratio of oxygen/carbon (O1s/C1s) of SBS at 0 h, 12 h, 48 h was 0.104, 0.177 and 0.224, respectively. This result implies that the oxidation and degradation reactions of SBS occur simultaneously. In addition, after thermal exposure for 12 h and 48 h, the relative concentration of C=C decreased to 1.02% and 0.43%, and that of C–OH(R), C=O and COOH(R) increased to 5.44% and 7.22%, 2.58% and 4.68%, and 0.82% and 2.56%, respectively. This illustrates that SBS in blends has a remarkable sensitivity to temperature and can further degrade into some oxygen-containing groups such as the hydroxyl and carboxyl group with the prolonging of aging time.

**Figure 5:**
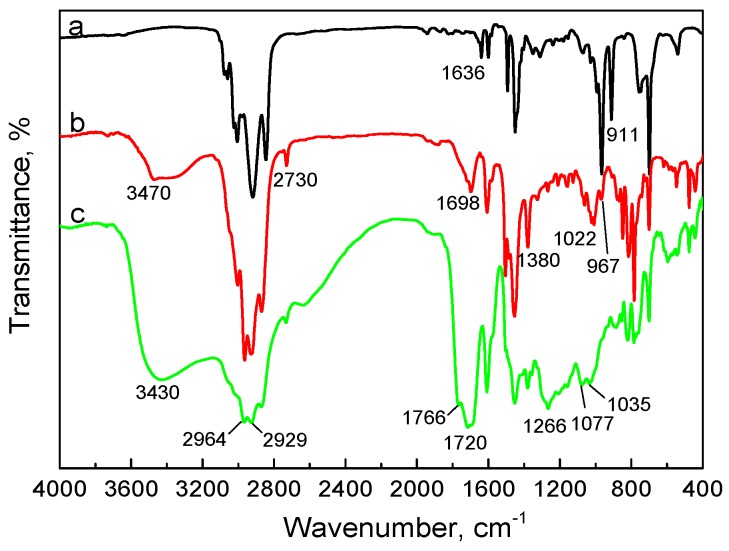
FTIR spectra of (**a**) neat SBS; (**b**) aged SBS exposure at 150 °C for 12 h with an oxygen flowrate of 1 L/min; (**c**) aged SBS exposure at 150 °C for 48 h with an oxygen flowrate of 1 L/min.

Moreover, the effect of aging time on the molecular structure of SBS, based on the FTIR spectra, is shown in Figure 5. It can be clearly observed that the characteristic bands attributed to –OH, C=O and C–O–C appear at 3470 cm^−1^, 1698 cm^−1^ and 1022 cm^−1^, and the characteristic band of C=C at 967 cm^−1^ clearly becomes weak in the spectrum of aged SBS (12 h), which is in accordance with the result of the XPS C1s spectra presented in Table 3. However, the spectrum of SBS after thermal aging for 48 h shows a significant change, especially around 3000 cm^−1^, the changed bands attributed to the asymmetric and symmetric stretching vibration of methylene illustrate that the more stable carbon–carbon single bond has suffered serious destruction under long-term thermal exposure. This is the reason why the relative concentration of C–C showed in Table 3 obviously decreases.

Due to the XPS and FTIR analysis above, the conclusion can be drawn that the extension of aging time can also accelerate the structural fracture and oxidation of strong bonds such as the C–C bond in SBS under 150 °C, which results in the production of the number of active oxygen-containing groups such as –OH, –COOH, etc.

### 3.3. Effect of Oxygen Concentration on the Molecular Structure of SBS

**Figure 6:**
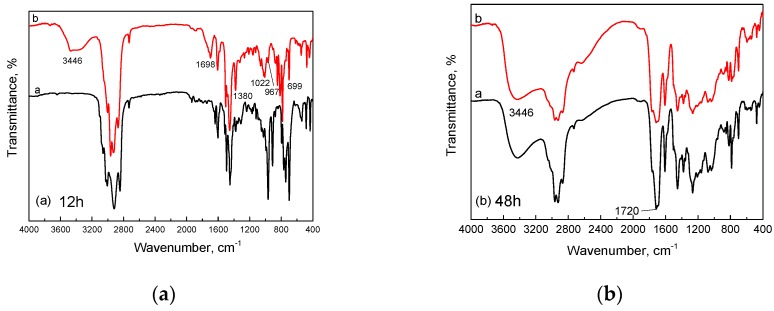
Effect of oxygen concentration on the molecular structure of the SBS in (**a**) short-term or (**b**) long-term aging (Oxygen flowrate: (**a**) 0.25 L/min; (**b**) 1 L/min; aging temperature: 150 °C).

The effect of oxygen concentration on the molecular structure of SBS in short- or long-term aging at 150 °C is shown in Figure 6. The oxygen concentration in the aging device was controlled by the change of the oxygen flowrate. In Figure 6a, it can be seen that when the oxygen flowrate increased from 0.25 L/min to 1 L/min, newly-formed characteristic absorption bands appeared at nearly 3446, 1698, 1380 and 1022 cm^−1^, attributed to –OH, C=O, –CH_3_ and C–O–C. It can also be seen that the characteristic band of C=C at 967 and 699 cm^−1^ became weak, which indicates that the increasing oxygen concentration can quicken the oxidative degradation of SBS in short-term aging (12 h). However, in Figure 6b, it can be seen that after thermo-oxidative aging for 48 h, the infra-red spectrum of SBS exposed to an oxygen flowrate of 1 L/min is very similar to that which was exposed to an oxygen flowrate of 0.25 L/min, illustrating that the rising oxygen content has little effect on the molecular structure of SBS in the long-term thermal aging procedure. The above results can be explained as being due to the rising oxygen concentration improving the collision probability between the polymeric radicals produced by high temperatures and the oxygen free radicals in the short-term aging procedure. As the aging time expanded to 48 h, the combination between polymeric radicals and oxygen free radicals almost finished, so the structure was not continuously oxidized with the increase in oxygen concentration.

### 3.4. Thermal Properties

**Figure 7:**
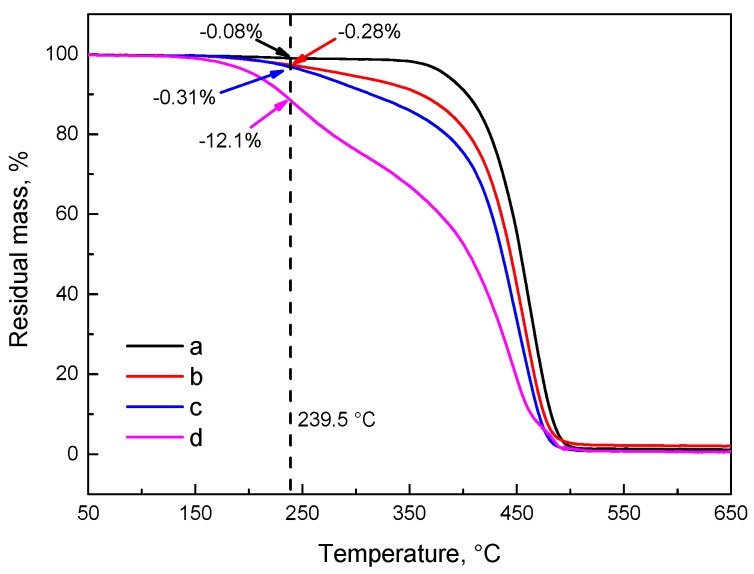
TG plots of (**a**) neat SBS; (**b**) aged SBS1 (150 °C × 3 h × 1 L/min); (**c**) aged SBS2 (180 °C × 3 h × 1 L/min); (**d**) aged SBS3 (150 °C × 48 h × 1 L/min).

**Figure 8:**
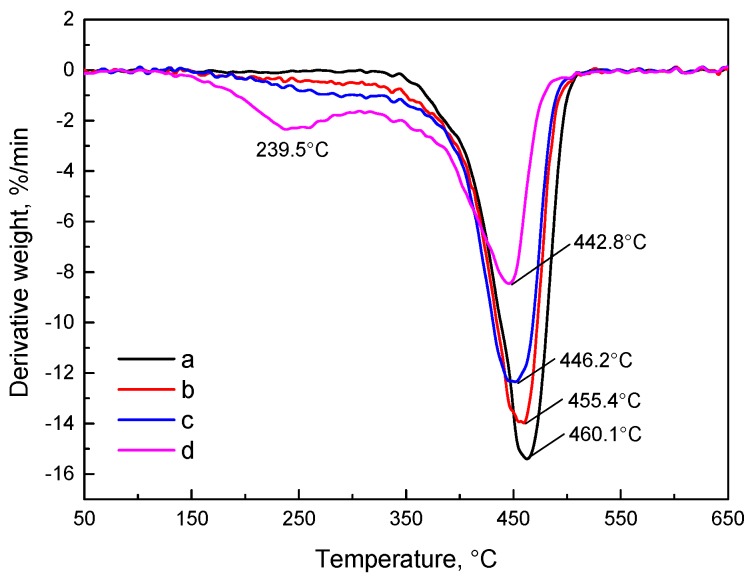
DTG plots of (**a**) neat SBS; (**b**) aged SBS1 (150 °C × 3 h × 1 L/min); (**c**) aged SBS2 (180 °C × 3 h × 1 L/min); (**d**) aged SBS3 (150 °C × 48 h × 1 L/min).

Figure 7 and Figure 8 show the TG and DTG curves of SBS after exposure to various thermo-oxidative aging conditions, and the characteristic parameters for neat and thermal-aged SBS are presented in Table 4. As seen in the plots of TG and DTG, the observed beginning and maximum decomposition temperatures shift to a lower temperature after thermal aging; especially in curve d, two peaks appeared in the DTG curves indicating that an adverse external environment can accelerate the decomposition of SBS. Comparing the plots b, c and d, the results can be obtained that, in terms of the thermal decomposition of SBS, the effects are more noticeable for prolonging the aging time than for improving the aging temperature in short-term aging. Besides, the mass loss of neat and thermal aged SBS at 239.5 °C reaches 0.08%, 0.28%, 0.31% and 12.1%, respectively. Also seen in Table 4, in the beginning stage, the initial decomposition temperature and decomposition rate of SBS decreases and increases, respectively, and the maximum decomposition temperature and rate both decrease as the aging level increases. This illustrates that an adverse external environment quickens the decomposition of SBS into low-molecular weight oxidative products. This result corresponds to the summary concerning the formation of C=O, C–O–C, –CH_3_, etc. and the weakness of C=C obtained from the FTIR and XPS C1s spectra.

### 3.5. Relation Between Thermal Properties and Oxygen-Containing Groups of Aged SBS

**Figure 9:**
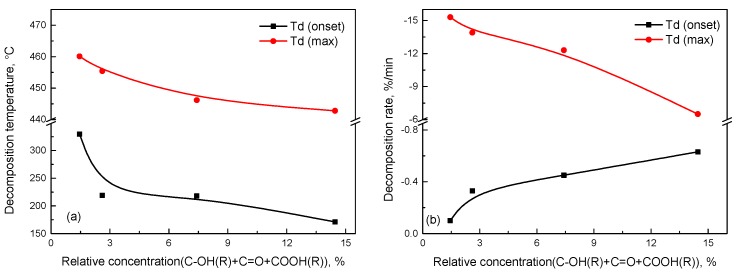
Relation between the relative concentration of oxygen-containing groups and (**a**) decomposition temperature or (**b**) decomposition rate based on XPS and TG-DTG.

From the discussion and analysis above, oxidation degradation is an important factor resulting in decreasing thermal properties. Figure 9 shows the effect of the relative concentration of oxygen-containing groups on the thermal properties of aged SBS. As can be seen in Figure 9a, the initial decomposition temperature quickly reduces to a low value (from 329.8 °C to 219.1 °C) and the maximum decomposition temperature (from 460.1 °C to 455.4 °C) slowly decreases when the relative concentration of oxygen-containing groups is less than 3%. Afterwards, the initial and the maximum decomposition temperature reduce slowly. The results indicate that part of the weak bond on the molecular structure of the SBS has been seriously damaged as the relative concentration of the oxygen-containing groups accumulates around 3%, which leads to the fast reduction of the initial thermal stability of the SBS. However, the reason for the slow decrease in the maximum decomposition temperature is that, when the relative concentration of the oxygen-containing groups is more than 3%, the residual weak bonds continue to be fractured and portions of the strong bonds start to break to produce more oxygen-containing groups.

In Figure 9b it can be observed that the initial decomposition rate increases and the maximum decomposition rate decreases with the rising concentration of oxygen-containing groups. It indicates that more low-molecular-weight substances that can be rapidly decomposed from the beginning appear with the increase of oxygen-containing groups. With regard to the decline of the maximum decomposition rate, it can be explained by the fact that the continued concentration of the oxygen-containing groups depends on the damage to the strong bond on the molecular structure of SBS. Compared with the seriously aged SBS, the slightly aged SBS has more bonds that can be destroyed at a relatively low temperature, thus, the maximum decomposition rate decreases with the accumulation of the oxygen-containing groups.

## 4. Conclusions

In the present work, the solvent naphtha, with a high boiling-point, was selected to substitute for bitumen in order to simulate the thermo-oxidative aging procedure of SBS in bitumen. The molecular structure and thermal properties of aged SBS in blends under various thermo-oxidative environments were also investigated, which can provide a basis for the structural reaction–reconstruction of aged SBS in bitumen. The relation between the thermal properties and oxygen-containing groups of aged SBS were further discussed. The following conclusions were obtained.

1. The FTIR and XPS spectra indicate that increments of aging temperature, aging time and oxygen concentration can accelerate the decomposition of tri-block structural SBS into bi-block structural SB- and the increasing concentration of oxygen-containing groups such as –OH, C=O, –COOH, etc. Increments of temperature or oxygen concentration have a significant effect on the structural destruction of SBS in short-term aging, while the rising oxygen concentration has no further effect on the molecular structure of SBS in long-term aging.
2. The TG and DTG results illustrate that a large number of low-molecular weight substances were formed in the adverse thermo-oxidative environment, and the initial and maximum decomposition temperature of aged SBS both decreased. With the rising degree of aging of the SBS, the initial decomposition rate gradually increases at the beginning of thermal weightlessness, and the decomposition rate slows down when in comparison with the neat SBS.
3. Based upon the relation of the relative concentration of oxygen-containing groups and the thermal properties of aged SBS, the initial thermal stability of SBS rapidly reduces as the relative concentration of the oxygen-containing groups accumulates around 3%. When the relative concentration of the oxygen-containing groups is more than 3%, the maximum decomposition temperature slowly decreases due to the difficult destruction of strong bonds on the molecular structure of aged SBS.

## Figures and Tables

**Table 1:** Effect of aging temperature on the relative functional group index of SBS.

Samples	Relative Functional Group Index				
	I_C=C_		I_C–OH(R)_		I_C=O_
	I_1639_	I_967_	I_3446_	I_1050~1300_	I_1695~1720_
Neat SBS	0.135	0.523	0	0.011	0
Aged SBS (150 °C)	0.128	0.509	0	0.029	0.009
Aged SBS (180 °C)	0.099	0.382	0.032	0.041	0.035

**Table 2:** Effect of aging temperature on the relative concentration of carbon-related groups of SBS.

Samples	Relative Concentration, %				
	C–C(H)	C=C	C–OH(R)	C=O	COOH(R)
	284.8 eV	285.5 eV	286.8 eV	288.0 eV	289.0 eV
Neat SBS	91.30	7.25	1.45	0	0
Aged SBS (150 °C)	90.22	7.17	2.25	0.24	0.12
Aged SBS (180 °C)	86.19	6.39	4.29	1.88	1.25

**Table 3:** Relative concentrations of carbon related to chemical groups at different aging times

Chemical State	Binding Energy (eV)	Relative Concentration (%)		
		Neat SBS	Aged SBS (12 h)	Aged SBS (48 h)
C–C(H)	284.8 ± 0.1	91.30	90.14	85.11
C=C	285.5 ± 0.1	7.25	1.02	0.43
C–O–H(R)	286.8 ± 0.1	1.45	5.44	7.22
C=O	288.0 ± 0.1	0	2.58	4.68
COOH(R)	289.0 ± 0.1	0	0.82	2.56

**Table 4:** Characteristic parameters for neat and thermo-aged SBS from TG and DTG plots.

Samples	Tonset, °C	Td (max), °C	Ronset, %/min	Rd (max), %/min
Neat SBS	329.8	460.1	−0.10	−15.3
Aged SBS1	219.1	455.4	−0.33	−13.9
Aged SBS2	217.9	446.2	−0.45	−12.3
Aged SBS3	171.1	442.8	−0.63	−8.39
